# Medical weight management protects against weight gain during the COVID‐19 pandemic

**DOI:** 10.1002/osp4.601

**Published:** 2022-03-16

**Authors:** Sarah R. Barenbaum, Katherine H. Saunders, Karina M. Chan, William J. Crowley, Ilana P. Redmond, Anthony J. Casper, Katie C. Hootman, Rajasekhar Ramakrishnan, Louis J. Aronne, Alpana P. Shukla

**Affiliations:** ^1^ Comprehensive Weight Control Center at Weill Cornell Medicine, Division of Endocrinology, Diabetes and Metabolism New York New York USA; ^2^ Institute of Human Nutrition Columbia University Irving Medicine Center New York New York USA; ^3^ NYU Long Island School of Medicine New York University Medical Center Mineola New York USA; ^4^ Division of General Internal Medicine Weill Cornell Medicine New York New York USA; ^5^ Clinical and Translational Science Center Weill Cornell Medicine New York New York USA; ^6^ Department of Pediatrics College of Physicians and Surgeons Columbia University New York New York USA

**Keywords:** antiobesity pharmacotherapy, COVID‐19 pandemic, obesity, weight gain, weight management

## Abstract

**Background:**

American adults have gained weight during the COVID‐19 pandemic. Little is known about how patients who are medically managed for overweight and obesity, including patients who are prescribed antiobesity pharmacotherapy, have fared.

**Objective:**

To assess the COVID‐19 pandemic's effect on weight, food choices, and health behaviors in patients receiving medical treatment for overweight or obesity.

**Methods:**

Adult patients treated at an urban academic weight management center between 1 May 2019 and 1 May 2020 were electronically surveyed between 23 February and 23 March 2021. The survey assessed changes in weight, eating, behaviors, and the use of antiobesity medications (AOMs) following issuance of social distancing/stay‐at‐home policies in March 2020.

**Results:**

In 970 respondents, median percent weight change for those taking AOMs was −0.459% [interquartile range −5.46%–(+3.73%)] compared to +2.33% [IQR −1.92%–(+6.52%)] for those not taking AOMs (*p* < 0.001). More participants achieved ≥5% weight loss if they were taking AOMs compared to those who were not (26.7% vs. 15.8%, *p* = 0.004), and weight gain ≥5% was also lower in those taking AOMs (19.8% vs. 30.3%, *p* = 0.004). Patients with pre‐pandemic BMI ≥30 kg/m^2^ taking AOMs experienced the greatest weight reduction, and there was greater weight loss associated with increased physical activity.

**Conclusions and Relevance:**

Medical weight management protected against weight gain during this period of the COVID‐19 pandemic. Increased physical activity, decreased alcohol intake, and use of AOMs were factors that contributed to this protective effect.

## 1 INTRODUCTION

For the first time since the Spanish flu pandemic in 1918 the majority of Americans were asked to practice social distancing and abide by stay‐at‐home orders, when possible, starting in March 2020 to slow the spread of COVID‐19. Forty‐five out of 50 states issued stay‐at‐home orders during this time, which led to widespread business closures and a significant increase in working from home.^1^ Other countries issued similar mandates, leading to major disruptions in national and international supply chains. These disruptions, both nationally and internationally, led to closures of restaurants, changes in access to certain foods at grocery stores, and changes in Americans' mental health and overall health behaviors (e.g., food choices, eating behaviors, physical activity, and alcohol consumption).^2^

During the COVID‐19 pandemic, many individuals have experienced significant weight gain.^3^, ^4^, ^5^ The American Psychological Association conducted a survey in February 2021 which found that 42% of American adult respondents reported weight gain since the start of the pandemic. Of this group, average reported weight gain was 29 pounds (with a median weight gain of 15 pounds) and 10% reported weight gain of more than 50 pounds.^6^ The cause of weight gain amongst American adults appears to be multifactorial and varied. While for some individuals weight gain was related to behavioral changes caused by stay‐at‐home orders, food scarcity, etc, for others the emergence of the global pandemic and the disruptions it caused also lead to changes in mental health which led to downstream behavioral changes.

Studies have highlighted a connection between stress and anxiety leading to negative effects on health behaviors and weight management during the COVID‐19 pandemic.^7^, ^8^, ^9^, ^10^ One study conducted in Rhode Island and Massachusetts evaluated the impact of stay‐at‐home orders at the outset of the pandemic in 99 individuals participating in an internet‐based weight loss program. This study found that greater stress was significantly associated with having less time to spend on weight‐loss efforts.^8^ Another survey study of individuals with overweight or obesity participating in two separate behavioral weight loss trials (n = 82) conducted during the stay‐at‐home mandate in Colorado concluded that most individuals experienced increased anxiety or stress. These participants reported difficulties adhering to recommended physical activity (68%) and to a prescribed diet (81%).^9^ Another survey study (n = 1198) of American adults found that anxiety, worry, and stress were associated with self‐reported increased eating.^10^

While studies have been published assessing the impact of COVID‐19 on mental health, behaviors, and weight among patients with overweight or obesity, little is known about how patients who are medically managed for their weight, which includes use of antiobesity pharmacotherapy, have fared. This study evaluated how the pandemic has affected weight, food choices, and health behaviors in patients receiving medical treatment for overweight or obesity at a large, urban, academic weight management center in the northeastern United States. The study's hypothesis was that the pandemic would have a negative impact on food choices and health behaviors but that medical weight management, including the use of antiobesity medications (AOMs) would protect against weight gain.

## 2 METHODS

Patients 18 and older treated for overweight or obesity between 1 May 2019 and 1 May 2020 at the Comprehensive Weight Control Center at Weill Cornell Medicine were identified via electronic health record and invited to complete an anonymous survey between February 23, and 23 March 2021. The survey assessed self‐reported changes in weight, eating, behaviors (including changes in physical activity), and the use of AOMs following issuance of social distancing/stay‐at‐home policies in March 2020. While all of the patients included in the study were being medically managed for their weight, specific treatment was highly individualized. All patients were counseled on diet and physical activity, but not all were prescribed AOMs.

Multivariable linear regression models were used to identify predictors of body weight change in this cross‐sectional study. Gender, age, ethnicity/race (white vs. non‐white), AOM use, pre‐pandemic BMI, cohabitation, urban setting, physical activity, and alcohol consumption were examined as predictors. Pairs of variables were evaluated for interaction, and identification of effect modification by AOM use and pre‐pandemic BMI led to stratification of the sample into analytical subgroups. Models were fit to each subgroup with sufficient sample size. Models were ranked by the corrected Akaike information criterion, the top‐ranked model was chosen, and likelihood ratio tests were used to confirm the chosen model improved the fit significantly (*p* < 0.05) compared to simpler models with the predictors in the chosen model. Data were analyzed in R (R Core Team, 2020) using standard R software functions (summary, aov, lm, estimable, dredge, ggplot, etc.) invoked by the cufunctions package.^11^, ^12^

## 3 RESULTS

Of the 4882 patients contacted for the survey, 970 individuals responded (response rate = 19.9%). Median pre‐pandemic BMI was 30.4 kg/m^2^ (interquartile range (IQR) 27.1–35), AOM use was common (n = 805, 83%; Table 1). Median weight change across the entire cohort was 0% [IQR −4.97% – (+4.32%)]. Median percent weight change for patients taking AOMs was −0.459% [IQR −5.46% – (+3.73%)] compared to +2.33% [IQR −1.92% – (+6.52%)] for those not taking AOMs (*p* < 0.001). More participants achieved ≥5% weight loss if they were taking AOMs compared to those who were not (26.7% vs. 15.8% respectively, *p* = 0.004), and weight gain ≥5% was lower in those taking AOMs (19.8% vs. 30.3% respectively, *p* = 0.004). Greater percent weight change was predicted by higher pre‐pandemic BMI.

**TABLE 1:** Demographics and self‐reported frequency of behaviors following the issuance of stay at home/social distancing policies in March 2020 in the analytical subgroups and full sample. [Some percentages, as for alcohol consumption, do not add up to 100 due to missing response data.]

	BMI ≥30 kg/m^2^, AOM	BMI ≥30 kg/m^2^, no AOM	BMI <30 kg/m^2^, AOM	BMI <30 kg/m^2^, no AOM	Full sample
N	417	98	388	67	970
Age (%)					
‐ Under 30	4.8	6.1	4.6	6.0	4.9
‐ 30–65	72.4	62.2	68.3	62.7	69.1
‐ Over 65	22.8	31.6	27.1	31.3	26.0
Female (%)	74.3	68.4	81.4	74.5	76.6
Non‐white (%)	14.9	15.3	10.8	19.4	13.6
Urban setting (%)	58.3	56.1	51.0	71.6	56.1
Living alone (%)	26.1	21.4	19.8	32.8	23.6
Eating home‐cooked meals (%)					
‐ About the same	17.5	18.4	19.1	9.0	17.6
‐ Less	9.4	12.2	8.8	7.5	9.3
‐ More	73.1	69.4	72.2	83.6	73.1
Alcohol consumption (%)					
‐ About the same	40.0	33.7	43.3	37.3	40.5
‐ Less	43.6	40.8	35.8	35.8	39.7
‐ More	15.1	20.4	20.4	26.9	18.6
Physical activity level (%)					
‐ About the same	17.3	14.3	15.7	13.4	16.1
‐ Much less	34.3	29.6	32.7	32.8	33.1
‐ Somewhat less	24.5	32.7	30.4	25.4	27.7
‐ Much more	8.9	8.2	8.5	11.9	8.9
‐ Somewhat more	15.1	14.3	12.6	16.4	14.1
Median pre‐pandemic BMI (kg/m^2^) (median and interquartile range)	34.7 (31.8–38.2)	34.7 (32.4–28.5)	26.6 (24.8–28.2)	27.1 (24.8–28.7)	30.4 (27.1–35)
Food choices (%)					
‐ About the same	27.1	19.4	33.8	23.9	28.8
‐ Healthier	38.6	33.7	22.9	32.8	31.4
‐ Unhealthier	34.3	46.9	43.3	43.3	39.8
Snacking (%)					
‐ About the same	35.5	27.6	38.7	40.3	36.3
‐ Less	26.9	13.3	18.3	14.9	21.2
‐ More	37.6	59.2	43.0	44.8	42.5
Comfort eating (%)					
‐ About the same	30.2	29.6	33.5	29.9	31.4
‐ Less	24.2	13.3	16.5	22.4	19.9
‐ More	45.6	57.1	50.0	47.8	48.7
Level of concern for next meals or groceries (%)					
‐ About the same	70.5	65.3	69.3	71.6	69.6
‐ Less	9.8	15.3	11.1	11.9	11.0
‐ More	19.7	19.4	19.6	16.4	19.4
Access to fresh produce (%)					
‐ About the same	75.3	78.6	79.6	76.1	77.4
‐ Less	14.9	11.2	11.9	10.4	13.0
‐ More	9.8	10.2	8.5	13.4	9.6
Level of control over what foods were in the house (%)					
‐ About the same	61.6	63.3	63.9	74.6	63.6
‐ Less	22.8	19.4	22.2	14.9	21.6
‐ More	15.6	17.3	13.9	10.4	14.7

**FIGURE 1:**
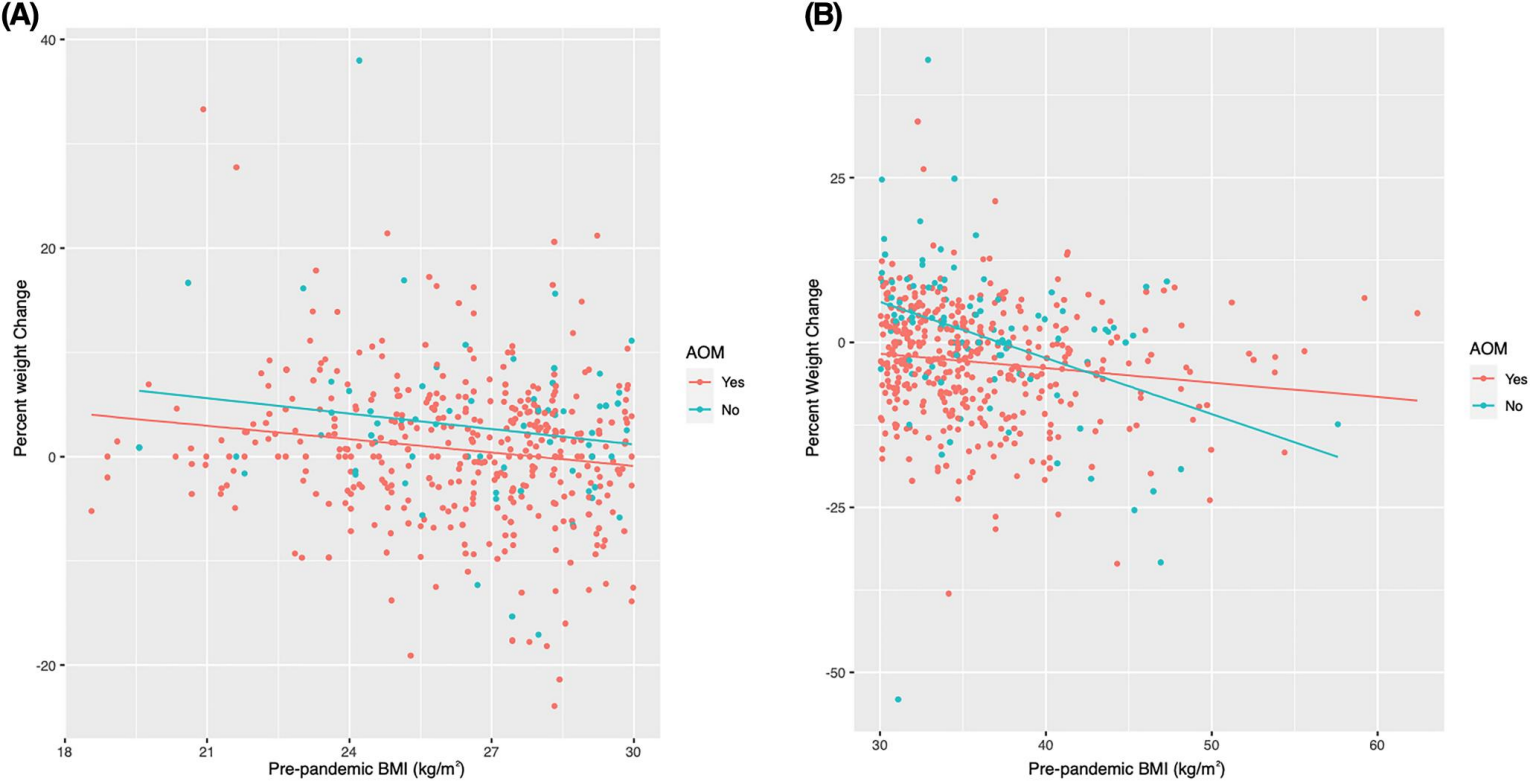
Percent weight change versus pre‐pandemic BMI, stratified by antiobesity medications (AOMs) usage. Panel A included participants with BMI <30 kg/m2 and panel B included participants with BMI ≥30 kg/m2

The relationship between percent weight change and pre‐pandemic BMI differed by AOM use and pre‐pandemic BMI (BMI <30 kg/m^2^ vs. BMI ≥30 kg/m^2^; Figure 1). To explore this relationship, the study population was divided into four analytical subgroups: 1. pre‐pandemic BMI ≥30 kg/m^2^ taking AOMs, 2. pre‐pandemic BMI <30 kg/m^2^ taking AOMs, 3. pre‐pandemic BMI ≥30 kg/m^2^ not taking AOMs, 4. pre‐pandemic BMI <30 kg/m^2^ not taking AOMs. Linear regression models were fitted to each cohort separately. Group 4 was not modeled due to insufficient sample size. Table 1 details the characteristics of the four subgroups and the entire cohort, and Figure 1 shows the percent weight change versus pre‐pandemic BMI, stratified by AOMs usage.

Patients with a pre‐pandemic BMI ≥30 kg/m^2^ taking AOMs experienced the greatest weight reduction. Greater weight loss was associated with increased physical activity, with a more pronounced effect at higher BMIs. For example, somewhat more physical activity was predicted to decrease body weight by 4.0% at BMI of 30 kg/m^2^ and by 19.4% at BMI of 50 kg/m^2^ (Table 2). Weight loss was also associated with reduced alcohol consumption and white race (Supp Table 1, Table 2). Among individuals with a pre‐pandemic BMI ≥30 kg/m^2^ not taking AOMs, weight loss was associated with higher pre‐pandemic BMI and increased physical activity (Supp Table 2, Table 2). Among individuals with pre‐pandemic BMI <30 kg/m^2^ taking AOMs, weight loss was associated with pre‐pandemic BMI and decreased alcohol consumption, whereas age over 65, decreased physical activity, non‐white status, and increased alcohol consumption were significantly associated with weight gain (Supp Table 3 and Supp Table 4). Decreased alcohol consumption predicted percent weight loss of 2.1% at BMI of 20 kg/m^2^ and 6.0% at BMI of 28 kg/m^2^ (Supp Table 4). Regression factors from Supp Tables [Link], [Link], [Link], [Link] were modeled to predict percent weight changes (Table 2 and Supp Table 4).

**TABLE 2:** Predicted percent weight change for three theoretical reference individuals with BMI ≥30 kg/m^2^ taking antiobesity medications (AOMs), based on Supp.Table 1 data. The reference is a white individual, with decreased alcohol consumption and unchanged physical activity level. And predicted percent weight change for three theoretical reference individuals with BMI ≥30 kg/m^2^ not taking AOMs, based on Supp.Table 2 data. The reference is an individual with same or increased level of physical activity

Reference individual	Pre‐pandemic BMI (kg/m^2^)	30	40	50
BMI ≥30 kg/m^2^ taking AOMs	Reference weight change (%)	−3.6	−6.7	−9.7
	Physical activity: Much less than usual	−1.2	−2.0	−2.8
	Physical activity: Somewhat less than usual	−3.2	−3.3	−3.4
	Physical activity: Somewhat more than usual	−4.0	−11.7	−19.4
	Physical activity: Much more than usual	−7.5	−13.6	−19.6
	Race: Non‐white	−1.4	−4.4	−7.5
	Alcohol consumption: Same or more	−1.4	−4.5	−7.5
BMI ≥30 kg/m^2^ not taking AOMs	Reference	+2.5	−6.8	−16.0
	Physical activity: Less than usual	+10.0	+0.8	−8.5

In the entire sample, 73.1% reported more home‐cooked meals and 60.8% reported less physical activity. There was greater weight loss with self‐reported increased physical activity, with a more pronounced effect at higher BMIs (Table 2). Increases in unhealthy food choices, snacking, and comfort eating (i.e., eating to make one feel better rather than eating due to hunger) were reported by 39.8%, 42.5% and 48.7%, respectively. Nearly 40% of patients reported decreased alcohol consumption (Table 1).

## 4 DISCUSSION

This study illustrates that medical weight management, particularly use of AOMs and increased physical activity, protects against weight gain and can also lead to weight loss during times of stress and environmental change. Increased physical activity, decreased alcohol intake, and use of AOMs were modifiable factors that contributed to this protective effect. Interestingly, the predicted impact of physical activity on weight loss increased exponentially at higher BMIs in those taking AOMs. Both directions of change in alcohol consumption during the pandemic have been reported in the literature.^13^, ^14^ In this study, the majority of respondents across all subgroups reported about the same or reduced alcohol consumption, a finding that may reflect the impact of lifestyle counseling or use of AOMs. While the survey was anonymous, this does not preclude some bias in self‐reported alcohol consumption.

This is the first study to investigate weight change in patients undergoing medical treatment including pharmacotherapy for overweight and obesity during the COVID‐19 pandemic. These findings offer unique insight into the utility of antiobesity pharmacotherapy and its interactions with health behaviors. While morbidity and mortality associated with the virus has decreased there is still uncertainty, anxiety and stress surrounding the pandemic. There are also semi‐permanent to permanent changes in Americans' daily routines which continue to impact overall health behaviors. Addressing both the mental health and behavioral health components is important to mitigate weight gain and promote overall health.

Limitations of this study include single center design with participants at different stages of weight‐loss/maintenance, and lack of a control group. The anthropometry data was also self‐reported and retrospective in nature. Intercurrent illness and unintentional weight loss could not be differentiated. Overall health and socioeconomic status of the participants were not controlled for.

Obesity is increasing rapidly in the United States and it has been projected that by 2030 nearly 50% of American adults over the age of 18 will have obesity.^15^, ^16^ While weight gain during the pandemic may seem trivial, research has shown that small changes in weight on vacations or during the holidays can lead to substantial and permanent weight gain over time.^17^, ^18^ It is therefore likely that weight gain during the pandemic may be lasting and may accelerate the rise of obesity in America. COVID‐19 has further highlighted the critical need to treat obesity as individuals with obesity are at higher risk for COVID‐19‐related morbidity and mortality.^19^, ^20^ Weight loss of 5%–10% among patients with obesity has been shown to be sufficient for clinically significant improvements in health.^21^, ^22^ Further research is needed to ascertain if this amount of weight loss could reduce the risk of severe disease from COVID‐19. However, obesity is a disease with serious consequences, as has been highlighted by the pandemic. Treating obesity, including prescribing antiobesity pharmacotherapy for those who qualify, should be a priority amongst clinicians as a powerful lever to decrease morbidity and all‐cause mortality related to obesity and the scores of weight‐related comorbidities.

## CONFLICTS OF INTEREST

Dr. Hootman reports being a consultant for Faeth Therapeutics, Inc.; and an educational program reviewer for PESI, Inc.

Dr. Saunders reports ownership/stock/management interest in Intellihealth.

Dr. Aronne reports receiving consulting fees from/and serving on advisory boards for Gelesis, Jamieson Wellness, Janssen Pharmaceuticals, Jazz Pharmaceuticals, Novo Nordisk, Pfizer, Real Appeal and Eli Lilly; receiving research funding from Allurion, Astra Zeneca, Gelesis, Janssen Pharmaceuticals, Novo Nordisk and Eli Lilly; having equity interests in Allurion, ERX Pharmaceuticals, Gelesis, Intellihealth, Jamieson Wellness, Myos Corp and Zafgen; and serving on a board of directors for Intellihealth and Jamieson Wellness.

The rest of the authors report no disclosures.

## AUTHOR CONTRIBUTIONS

*Concept and design:* Barenbaum, Shukla, Saunders, Redmond, Hootman, Aronne; *Acquisition, analysis, or interpretation of data:* All authors; *Drafting of the manuscript:* Barenbaum, Shukla, Saunders, Chan; *Critical revision of the manuscript for important intellectual content:* All authors; *Statistical analysis:* Chan, Crowley, Ramakrishnan; *Administrative, technical, or material support:* Casper; *Study supervision*: Shukla, Saunders.

## Supporting information

Table S1

Table S2

Table S3

Table S4

## References

[1] FINRA. State “Shelter‐in‐place” and “stay‐at‐home “orders. Published2020 . https://www.finra.org/rules‐guidance/key‐topics/covid‐19/shelter‐in‐place. Accessed May 10, 2021.

[2] Robinson E , Gillespie S , Jones A . Weight‐related lifestyle behaviours and the COVID‐19 crisis: an online survey study of UK adults during social lockdown. Obesity Science and Practice. 2020;6:735‐740. 33354349 10.1002/osp4.442 PMC7746963

[3] Lin A , Vittinghoff E , Olgin J , Pletcher MJ , Marcus GM . Body weight changes during pandemic‐related shelter‐in‐place in a longitudinal cohort study. JAMA Netw Open. 2021;4(3):e212536. 10.1001/jamanetworkopen.2021.2536 33749764 PMC7985720

[4] Bhutani S , vanDellen M , Cooper J . Longitudinal weight gain and related risk behaviors during the COVID‐19 pandemic in adults in the US. Nutrients. February. 2021;13(2):671. 10.3390/nu13020671 PMC7922943 33669622

[5] Zeigler Z . COVID‐19 self‐quarantine and weight gain risk factors in adults. Current Obesity Reports. Jul 2021 12:1‐11. 34251647 10.1007/s13679-021-00449-7 PMC8273568

[6] American Psychological Association . Stress in America: One Year Later, A New Wave of Pandemic Health Concerns. March 2021. Found at: https://www.apa.org/news/press/releases/stress/2021/sia‐pandemic‐report.pdf

[7] Caldwell A , Thomas E , Rynders C , et al. Improving lifestyle obesity treatment during the COVID‐19 pandemic and beyond: new challenges for weight management. Obesity Science and Practice. 2021:1‐13. 10.1002/osp4.540 PMC8441901 34540266

[8] Pellegrini C , Webster J , Hahn K , Leblond TL , Unick JL . Relationship between stress and weight management behaviors during the COVID‐19 pandemic among those enrolled in an internet program. Obesity Science and Practice. 2021;7:129‐134. 33680497 10.1002/osp4.465 PMC7909591

[9] Caldwell A , Thomas E , Rynders C , et al. Improving lifestyle obesity treatment during the COVID‐19 pandemic and beyond: new challenges for weight management. Obesity Science and Practice. 2021:1‐13. 10.1002/osp4.540 PMC8441901 34540266

[10] Himmelstein M , Beaver J , Gilman T . Anxiety and stress over COVID‐19 pandemic associated with increased eating. Obesity Science and Practice. 2021:1‐14. 10.1002/osp4.576 PMC9159555 35664251

[11] R Core Team . R: A Language and Environment for Statistical Computing. R Foundation for Statistical Computing; 2020.

[12] Holleran S , Ramakrishnan R . Cufunctions, a Package to Facilitate Statistical Analyses in R; 2021. http://biomath.net/cufunctions.html

[13] Naughton F , Ward E , Khondoker M , et al. Health behaviour change during the UK COVID‐19 lockdown: findings from the first wave of the C‐19 health behaviour and well‐being daily tracker study. Br J Health Psychol. 2021;26(2):624‐643. 33410229 10.1111/bjhp.12500 PMC9291054

[14] Zupo R , Castellana F , Sardone R , et al. Preliminary trajectories in dietary behaviors during the COVID‐19 pandemic: a public health call to action to face obesity. Int J Environ Res Public Health. 2020;17(19):7073. 10.3390/ijerph17197073 PMC7579065 32992623

[15] Hales C , Carroll M , Frayar C , et al. Prevalence of Obesity and Severe Obesity Among Adults: United States, 2017‐2018. U.S. Department of Health and Human Services; NCHS Data Brief https://www.cdc.gov/nchs/data/databriefs/db360‐h.pdf 32487284

[16] Ward Z , Bleich S , Cradock A , et al. Projected U.S. State‐level prevalence of adult obesity and severe obesity. New England Journal of Medicine; December. 2019;381:25. 10.1056/NEJMsa1909301 31851800

[17] Yanovski J , Yanovski S , Sovik K , Nguyen TT , O'Neil PM , Sebring NG . A prospective study of holiday weight gain. N Engl J Med. 2000;342:861‐867. 10727591 10.1056/NEJM200003233421206 PMC4336296

[18] Schoeller D . The effect of holiday weight gain on body weight. Physiol Behav. 2014;134:66‐69. 24662697 10.1016/j.physbeh.2014.03.018

[19] Goyal P , Choi J , Pinheiro L , et al. Clinical characteristics of covid‐19 in New York city. N Engl J Med;Correspondence to the Editor. 4/17/2020 10.1056/NEJMc2010419 PMC7182018 32302078

[20] Underlying Medical Conditions Associated with High Risk for Severe COVID‐19: Information for Healthcare Providers. https://www.cdc.gov/coronavirus/2019‐ncov/hcp/clinical‐care/underlyingconditions.html Accessed 9/20/2021.

[21] Wing RR , Lang W , Wadden TA , et al. Benefits of modest weight loss in improving cardiovascular risk factors in overweight and obese individuals with type 2 diabetes. Diabetes Care. 2011;34:1481‐1486. 21593294 10.2337/dc10-2415 PMC3120182

[22] Magkos F , Fraterrigo G , Yoshino J , et al. Effects of moderate and subsequent progressive weight loss on metabolic function and adipose tissue biology in humans with obesity. Cell Metab 2016;23:591‐601. 26916363 10.1016/j.cmet.2016.02.005 PMC4833627

