# Supplementary material for: Medical weight management protects against weight gain during the COVID‐19 pandemic
Source: Obes Sci Pract. 2022 Mar 16;8(5):682–7. doi: 10.1002/osp4.601 (PMC9535662; doi:10.1002/osp4.601)
Supplement: Supplementary file 1 — Table S1 [file OSP4-8-682-s003.docx]

Supp. Table 1. Results of multivariable linear regressions predicting percent weight change in individuals with BMI ≥30 kg/m^2^ taking AOMs. Variable levels reflect the survey verbiage used to query behavior changes that occurred after March 2020.

|  | Estimate | Standard Error | p-value |
| --- | --- | --- | --- |
| Intercept | -3.635 | 1.52 | 0.02 |
| Alcohol consumption: Same or more | +2.212 | 0.79 | 0.01 |
| Physical Activity: Much less than usual | +2.427 | 1.72 | 0.16 |
| Physical Activity: Much more than usual | -3.820 | 2.58 | 0.14 |
| Physical Activity: Somewhat less than usual | +0.479 | 1.82 | 0.79 |
| Physical Activity: Somewhat more than usual | -0.383 | 2.21 | 0.86 |
| Pre-Pandemic BMI 30 kg/m^2^ | -0.305 | 0.18 | 0.10 |
| Race: Non-white | +2.275 | 1.09 | 0.04 |
| Interaction: pre-pandemic BMI and |  |  |  |
| Physical Activity: Much less than usual | +0.224 | 0.22 | 0.30 |
| Physical Activity: Much more than usual | -0.305 | 0.31 | 0.32 |
| Physical Activity: Somewhat less than usual | +0.295 | 0.23 | 0.21 |
| Physical Activity: Somewhat more than usual | -0.463 | 0.32 | 0.15 |
