# Supplementary material for: Medical weight management protects against weight gain during the COVID‐19 pandemic
Source: Obes Sci Pract. 2022 Mar 16;8(5):682–7. doi: 10.1002/osp4.601 (PMC9535662; doi:10.1002/osp4.601)
Supplement: Supplementary file 2 — Table S2 [file OSP4-8-682-s001.docx]

Supp. Table 2. Results of multivariable linear regressions predicting percent weight change in individuals with BMI $\geq$ 30 kg/m^2^ not taking AOMs.

|  | Estimate | Standard Error | p-value |
| --- | --- | --- | --- |
| Intercept | +2.532 | 1.91 | 0.19 |
| Physical Activity: Less than usual | +7.510 | 1.89 | <0.01 |
| Pre-Pandemic BMI - 30 | -0.929 | 0.18 | <0.01 |
