# Supplementary material for: Medical weight management protects against weight gain during the COVID‐19 pandemic
Source: Obes Sci Pract. 2022 Mar 16;8(5):682–7. doi: 10.1002/osp4.601 (PMC9535662; doi:10.1002/osp4.601)
Supplement: Supplementary file 3 — Table S3 [file OSP4-8-682-s002.docx]

Supp. Table 3. Results of multivariable linear regressions predicting percent weight change in individuals with BMI < 30 kg/m^2^ taking AOMs. Variable levels reflect the survey verbiage used to query behavior changes that occurred after March 2020.

|  | Estimate | Standard Error | p-value |
| --- | --- | --- | --- |
| Intercept | -0.836 | 1.84 | 0.65 |
| Age: 30-65 | +2.151 | 1.56 | 0.17 |
| Age: Over 65 | +3.783 | 1.66 | 0.02 |
| Alcohol consumption: Less than usual | -1.220 | 0.75 | 0.10 |
| Alcohol consumption: More than usual | +2.508 | 0.89 | <0.01 |
| Physical Activity: Less than usual | +3.330 | 0.94 | <0.01 |
| Physical Activity: More than usual | -1.231 | 1.11 | 0.27 |
| Pre-Pandemic BMI - 20 | -0.487 | 0.14 | <0.01 |
| Race: Non-white | +2.543 | 1.05 | 0.02 |
