# Supplementary material for: Medical weight management protects against weight gain during the COVID‐19 pandemic
Source: Obes Sci Pract. 2022 Mar 16;8(5):682–7. doi: 10.1002/osp4.601 (PMC9535662; doi:10.1002/osp4.601)
Supplement: Supplementary file 4 — Table S4 [file OSP4-8-682-s004.docx]

Supp. Table 4. Predicted percent weight change for three theoretical reference individuals with BMI < 30 kg/m^2^ taking AOMs, based on Supp.Table 3 data. The reference is a white individual, aged < 30 years, with unchanged levels of alcohol consumption and physical activity.

| Pre-pandemic BMI (kg/m^2^) | 20 | 25 | 28 |
| --- | --- | --- | --- |
| Reference | -0.8 | -3.3 | -4.7 |
| Alcohol consumption: Less than usual | -2.1 | -4.5 | -6.0 |
| Alcohol consumption: More than usual | +1.7 | -0.8 | -2.2 |
| Physical Activity: Less than usual | +2.5 | +0.1 | -1.4 |
| Physical Activity: More than usual | -2.1 | -4.5 | -6.0 |
| Race: Non-white | +1.7 | -0.7 | -2.2 |
| Age: 30-65 | +1.3 | -1.1 | -2.6 |
| Age: Over 65 | +3.0 | +0.5 | -1.0 |
